# Cure of Strangulated Hernia by Aspiration

**Published:** 1872-10

**Authors:** 


### Cure of Strangulated Hernia by Aspiration.

M. DEMARQUAY communicates to *Le Mouvement Medical* a case of successful reduction of, and recovery from, strangulated congenital inguinal hernia by means of aspiration. A young man, 21 years of age, after fatigue during the 5th of May, while on a visit to his friends at Versailles, was suddenly seized with colic, attended by frequent vomiting; a tumour of considerable size at the same time had formed in the left groin. On the following day these symptoms continuing, he was taken to the surgical *servir* of the *Maison de Sante,* where the *interne,* having unsuccessfully employed the taxis, applied a bladder filled with ice, and waited for the morrow. On the morning of the 7th the patient was seen by M. Demarquay. He was then feverish and thirsty; the tumour voluminous, and it was seen that its precise nature was that of strangulated congenital hernia, of which M. Demarquay states he had never previously successfully treated a case by operation. The employment of the taxis having failed, the patient was put under the influence of an anæsthetic, and again the same measure taken with no better result. It was then determined to employ aspiration. A fine trocar was inserted at the centre of the tumour, and by means of the aspirator of M. Potain 120 grammes of fluid were extracted from the intestine. without counting the gas. The tumour instantly collapsed; the trocar was removed; an interval of a few minutes allowed to elapse before further interference took place. No swelling took place nor was there indication that further escape of gas or liquid from the intestine was going on; the taxis was again very gently applied, pressure was made very gently above and below the tumour, and the intestine felt to glide back into the cavity of the abdomen. The patient was subsequently treated by perfect quiet, with low diet, opium in small doses being given, and, beyond the occurrence of some degree of inflammation in the testicles from the pressure and handling it had undergone, no accident took place.—*The Doctor.*

